# The dynamics of dynamic time warping in fMRI data: A method to capture inter-network stretching and shrinking via warp elasticity

**DOI:** 10.1162/imag_a_00187

**Published:** 2024-06-03

**Authors:** Sir-Lord Wiafe, Ashkan Faghiri, Zening Fu, Robyn Miller, Adrian Preda, Vince D. Calhoun

**Affiliations:** Tri-Institutional Center for Translational Research in Neuroimaging and Data Science (TReNDS), Georgia State University, Georgia Institute of Technology, Emory University, Atlanta, GA, United States; University of California, Irvine, Irvine, CA, United States

**Keywords:** dynamic time warping (DTW), coupling stretching/shrinking, warp elasticity, warp deviation, functional magnetic resonant imaging (fMRI)

## Abstract

In neuroimaging research, understanding the intricate dynamics of brain networks over time is paramount for unraveling the complexities of brain function. One approach commonly used to explore the dynamic nature of brain networks is functional connectivity analysis. However, while functional connectivity offers valuable insights, it fails to consider the diverse timescales of coupling between different brain regions. This gap in understanding leaves a significant aspect of brain dynamics unexplored in neuroimaging research. We propose an innovative approach that delves into the dynamic coupling/connectivity timescales of brain regions relative to one another, focusing on how brain region couplings stretch or shrink over time, rather than relying solely on functional connectivity measures. Our method introduces a novel metric called “warping elasticity,” which utilizes dynamic time warping (DTW) to capture the temporal nuances of connectivity. Unlike traditional methods, our approach allows for (potentially nonlinear) dynamic compression and expansion of the time series, offering a more intricate understanding of how coupling between brain regions evolves. Through the adaptive windows employed by the DTW method, we can effectively capture transient couplings within varying connectivity timescales of brain network pairs. In extensive evaluations, our method exhibits high replicability across subjects and diverse datasets, showcasing robustness against noise. More importantly, it uncovers statistically significant distinctions between healthy control (HC) and schizophrenia (SZ) groups through the identification of warp elasticity states. These states are cluster centroids, representing the warp elasticity across subjects and time, offering a novel perspective on the dynamic nature of brain connectivity, distinct from conventional metrics focused solely on functional connectivity. For instance, controls spend more time in a warp elasticity state characterized by timescale stretching of the visual domain relative to other domains, suggesting disruptions in the visual cortex. Conversely, patients show increased time spent in a warp elasticity state with stretching timescales in higher cognitive areas relative to sensory regions, indicative of prolonged cognitive processing of sensory input. Overall, our approach presents a promising avenue for investigating the temporal dynamics of brain network interactions in functional magnetic resonance imaging (fMRI) data. By focusing on the elasticity of connectivity timescales, rather than adhering to functional connectivity metrics, we pave the way for a deeper understanding of neuropsychiatric disorders in neuroscience research.

## 1 Introduction

There is a growing body of evidence supporting the notion that the brain functions as a highly intricate interconnected system (Achard et al., 2006;Calhoun et al., 2014;Hutchison et al., 2013;Iraji et al., 2020;Menon, 2011;O’Neill et al., 2018;Sporns, 2016;Van den Heuvel & Sporns, 2013;Yaesoubi et al., 2015). In the field of neuroimaging, researchers typically explore this interconnectedness through functional connectivity (FC) analysis. FC investigates and characterizes patterns of coordinated activity within the brain by assessing temporal similarities among different brain regions. This approach has been extensively applied using functional magnetic resonance imaging (fMRI) data (Biswal et al., 1995;Chang & Glover, 2010;Fox et al., 2005). Traditional approaches such as Pearson correlation or partial correlation analyze static connectivity. Functional network connectivity (FNC) studies temporal similarity among overlapping whole-brain FC networks (Jafri et al., 2008). Recent advancements enabled the investigation of time-varying changes in FC over time (Allen et al., 2014;Chang & Glover, 2010;Hutchison et al., 2013;Sakoğlu et al., 2010), termed dynamic functional connectivity (Hutchison et al., 2013), time-varying functional connectivity (TVFC) (Lurie et al., 2020), or time-resolved functional connectivity (trFC) (Calhoun et al., 2014;Fukushima et al., 2018;Hutchison et al., 2013;Iraji et al., 2020;Lurie et al., 2020). The term “time-resolved functional network connectivity” (trFNC) is typically used in the context of studying trFC within intrinsic networks derived from fMRI data. This typically involves techniques such as independent component analysis to estimate these intrinsic networks (Calhoun et al., 2001;Du et al., 2020).

Several studies have studied trFC/trFNC utilizing methods such as sliding window Pearson correlation (SWPC) (Allen et al., 2014;Faghiri et al., 2023;Leonardi & Van De Ville, 2015;Sakoğlu et al., 2010), phase synchrony (PS), (Glerean et al., 2012;Honari et al., 2021;Pedersen et al., 2018), wavelet coherence (Chang & Glover, 2010;Yaesoubi et al., 2015), and coactivation patterns (Eickhoff et al., 2011;Liu et al., 2018). SWPC pairs the sample Pearson correlation estimator with a sliding window to estimate temporally resolved Pearson correlation. This approach offers simplicity and computational efficiency. Wavelet cross-coherence is an effective method for estimating coherence, which is essentially the frequency analogue to correlation, along with phase lags (Chang & Glover, 2010;Torrence & Compo, 1998), which may occur in fMRI (Friston, 2009). Despite the advantages of wavelet coherence, SWPC remains popular due to its established nature and ease of use, providing valuable insights into dynamic brain function.

While functional connectivity analysis provides useful information about brain function and its connectivity, traditional methods such as SWPC have notable limitations in their study of brain interconnectedness. It does not account for the varying time lag between blood-oxygenation-level-dependent fMRI signals, disregarding potential variations due to factors such as brain region location and neural processes (Friston, 1994;Lindquist et al., 2009). Moreover, SWPC fails to consider induced FC changes from physiological noise and motion artifacts over time, leading to potentially inaccurate connectivity estimates (Rangaprakash et al., 2018). Additionally, SWPC’s reliance on fixed window types and sizes influences connectivity results significantly (Hutchison et al., 2013).

Dynamic time warping (DTW) offers a solution to some of these issues by aligning time series with variable temporal shifts, improving static functional connectivity estimation (Linke et al., 2020;Meszlényi et al., 2017;Philips et al., 2021).Meszlényi et al. (2017)demonstrated DTW’s superior ability to capture connectivity amidst global noise and transient interactions compared with static correlation, also showing its reduced sensitivity to global signal regression, which shifts the distribution of Pearson correlation coefficients (Murphy et al., 2009).

DTW is a dynamic programming algorithm that aligns pairs of time series to each other obtaining a measure of similarity by allowing adaptive “elastic” transformations of time series pairs to find similar patterns (Senin, 2008). This measure of similarity is obtained by computing the minimum distance cost of alignment between pairs of time series, within an allowable window of the signal pairs (Sakoe & Chiba, 1978). The distance cost of DTW is obtained by finding the minimal sum of the distance (e.g., Euclidean, city-block) costs of warping two signals to each other.Meszlényi et al. (2017)used the distance cost from alignment pairs of time series as the functional connectivity measure against correlation coefficients (Linke et al., 2020). They focused on comparing the robustness of DTW distances and correlation coefficients to global regression signal, and the test–retest reliability of two measures in three cohorts of children, adolescents, and adults with and without autism spectrum disorders. They show that DTW outperforms classic correlation coefficients in these studies and show that more group differences could be detected from DTW than correlation.

While the DTW study performed byMeszlényi et al. (2017)is helpful, it provides a single measure of similarity between two brain networks and not the underlying nature of the complexities of their couplings. Also, methods that estimate trFC often overlook the crucial possibility of changes in coupling timescales between brain region pairs. FC methods such as SWPC are limited in their ability to capture variations in coupling timescales between brain regions. They fail to account for scenarios where one brain region may exhibit dynamic coupling with another at different temporal resolutions. For instance, consider a scenario where 10 seconds of one brain network or region is dynamically coupled to 15 seconds of another brain network or region. In such cases, FC measures such as SWPC and instantaneous methods such as instantaneous phase synchrony would fail to capture these intricate dynamic coupling resolutions.

In contrast, DTW has emerged as a promising approach for FC analysis, as it effectively allows for nonlinear mapping and dynamic elastic transformations between time series pairs. Building upon this capability, DTW can be extended to study the stretching and shrinking of coupling or connectivity timescales between brain region time series. We introduce an innovative approach, utilizing the adaptive “elastic” and varying windows provided by DTW, to dissect the intricate interplay between brain regions. Central to our methodology is the concept of “warp deviation,” which we define as the differential in warping paths, produced from DTW, between time series pairs. This concept allows us to estimate the relative timings of brain networks. Building upon warp deviation, we further conceptualize “warp elasticity”—a novel metric representing the degree of temporal expansion (stretching) or contraction (shrinking) of brain regions couplings relative to each other. By computing the derivative of warp deviation, we can effectively quantify warp elasticity, thus illuminating dynamic fluctuations in brain connectivity timescales at specific moments. Our approach represents a significant advancement in neuroimaging studies by providing a nuanced understanding of asymmetric patterns, where stretching of the temporal coupling in one brain region (indicating high cognitive processing) corresponds to shrinking of the temporal coupling (potentially indicating slower processing) in another. Here, we hypothesize that temporal stretching in the connectivity timescale of one brain region, potentially indicative of high-level processing, is counterbalanced by temporal shrinking in the connectivity timescale of another, suggesting low-level processing of brain networks. This methodology not only promises to unravel the complex dynamics of brain connectivity but also provides crucial insights into the neurological underpinnings of these processes.

We demonstrate the replicability of the warp elasticity across subject samples and datasets and demonstrate its robustness to noise. Additionally, we observed statistically significant differences between schizophrenia (SZ) and healthy control (HC) groups in the dynamics of the clusters obtained from the warp elasticity. These findings highlight the potential of DTW as a valuable tool for studying brain connectivity dynamics.

In our study, we utilized independent component analysis (ICA) to extract intrinsic networks from fMRI data. These brain networks represent distinct functional patterns within the brain. Subsequently, we employed DTW to analyze these networks and computed the warp elasticity for each pair of brain networks.

## 2 Method

### 2.1 Dynamic time warping

DTW is an algorithm that is employed to find an optimal alignment between two time series signals by stretching both of them in time while minimizing the distance between corresponding points in the two time series signals (Paliwal et al., 1982;Sakoe & Chiba, 1978). This involved first computing a distance matrix between the two time series signals, where each entry represents the distance between two points in the two series. We refer to this matrix as the index-by-index distance matrix since the distance is computed for each index pair between the time series pair. The matrix is then traversed from the last index pair to find the optimal path that minimizes the cumulative distance between corresponding points in the two series. The term used to represent the total accumulated distance is the “distance cost.” The choice of distance metric for calculating the distance cost in DTW can include options such as Euclidean distance, city-block distance, symmetric Kullback-Leibler divergence, and squared Euclidean distance. In our study, we opted for the Euclidean distance due to its simplicity and computational efficiency. Furthermore, our decision was guided by previous studies in the field, which also utilized the Euclidean distance metric. This choice ensures consistency with prior research in fMRI studies (Linke et al., 2020;Meszlényi et al., 2017;Philips et al., 2021). The following steps outline the procedure for executing the DTW algorithm. LetXandYbe discrete time series of lengthNandM,respectively.

Steps:

1. Compute the pairwise Euclidean distance matrix between all pairs of points inXandY.
2. Initialize the cumulative distance matrixDwith all entries set to infinity, except forD[0,0]=0.
3. For each indexiin the range[1,N]and each index*j*in the range[1,M], compute the cumulative distanceD[i,j]as follows:
  $$\begin{array}{l} D\left[i,j\right]=d\left(X\left[i\right],Y\left[j\right]\right)\\ \;\;\;\;\;\;\;\;\;\;\;\;\;\;+min\left(D\left[i-1,j\right],D\left[i,j-1\right],D\left[i-1,j-1\right]\right) \end{array}$$,
  whered(X[i],Y[j])is the distance between thei-th point inXand the*j*-th point inY.
4. The optimal warping path is then found by backtracking throughDfromD[N,M]toD[0,0], following the path with the lowest cumulative distance.
5. The warping path can be used to align the two time series signals by warping one of them according to the indices in the path.

### 2.2 Warping path

Prior to the DTW algorithm, each time series is assumed to be mapped to one another by their consecutive indexes. For two time series, TS-A and TS-B, of equal lengths, index 1 of TS-A is mapped to index 1 of TS-B, index 2 of TS-A and TS-B are mapped together and continually maps in parallel to one another till the end as shown inFigure 1(A). In addition to the distance cost, the DTW algorithm also provides another output: the optimal warping path. The optimal warping path is determined by following the path of the lowest accumulated distance within the index-by-index distance matrix. It is commonly referred to as the “warping path” of DTW and is shown inFigure 2with the green line within the heatmap matrix. This warping path is essentially a sequence of indexes from the time series pair, depicting how each time series is stretched or relatively shrunk. This path effectively captures the extent of stretching and relative shrinking needed to align the time series pair. It is important to note that we use the term “relative shrinking” because DTW does not compress time series, it only stretches them. However, when one time series is stretched, it appears as a shrink relative to the time series that remains unstretched. It is important to clarify that the term “stretching” in this context refers to the amount of adjustment needed by the DTW algorithm to align time series accurately. This should not be confused with the genuine stretching or shrinking occurring within the time series data, which is what we aim to estimate and understand through our methodology.

**Fig. 1.**
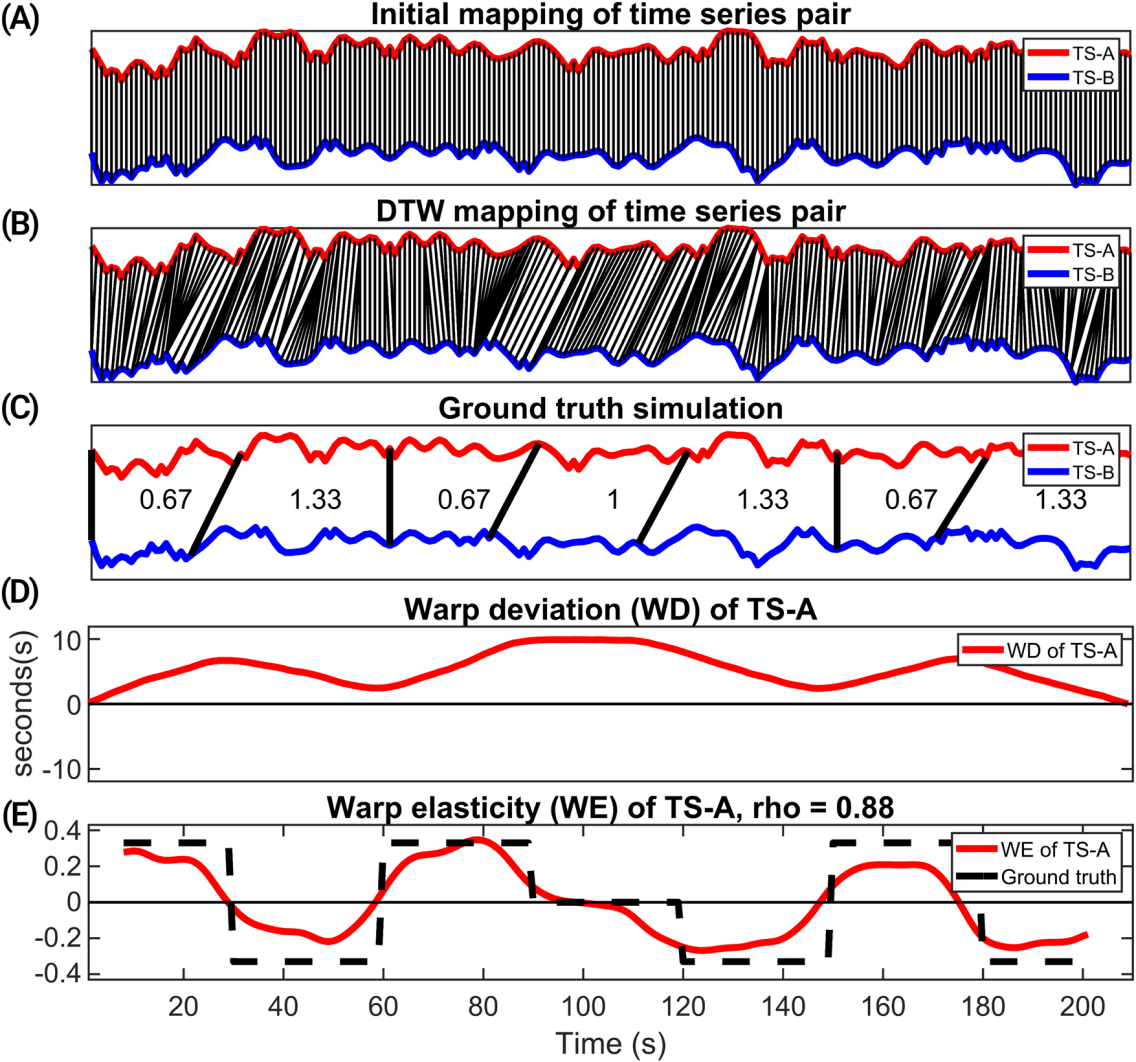
The application of the DTW algorithm on two signals, TS-A and TS-B. These signals, comprising 210 time points, were generated as bandlimited random time series, with TS-B being a resampled version of TS-A. (A) The temporal alignment of TS-A and TS-B before undergoing the DTW procedure. In contrast, (B) displays their alignment after applying DTW. (C) The sampling factor segments of TS-A applied systematically to create TS-B. Here, a sampling factor above 1 indicates stretching in TS-B, a factor less than 1 signifies shrinking of TS-B, and a factor of 1 means no stretching or shrinking. (D) The warp deviation (WD) of TS-A in seconds (s). (E) Comparison of the ground truth stretching and shrinking factor with the warp elasticity’s (WE) estimate of TS-A.

**Fig. 2.**
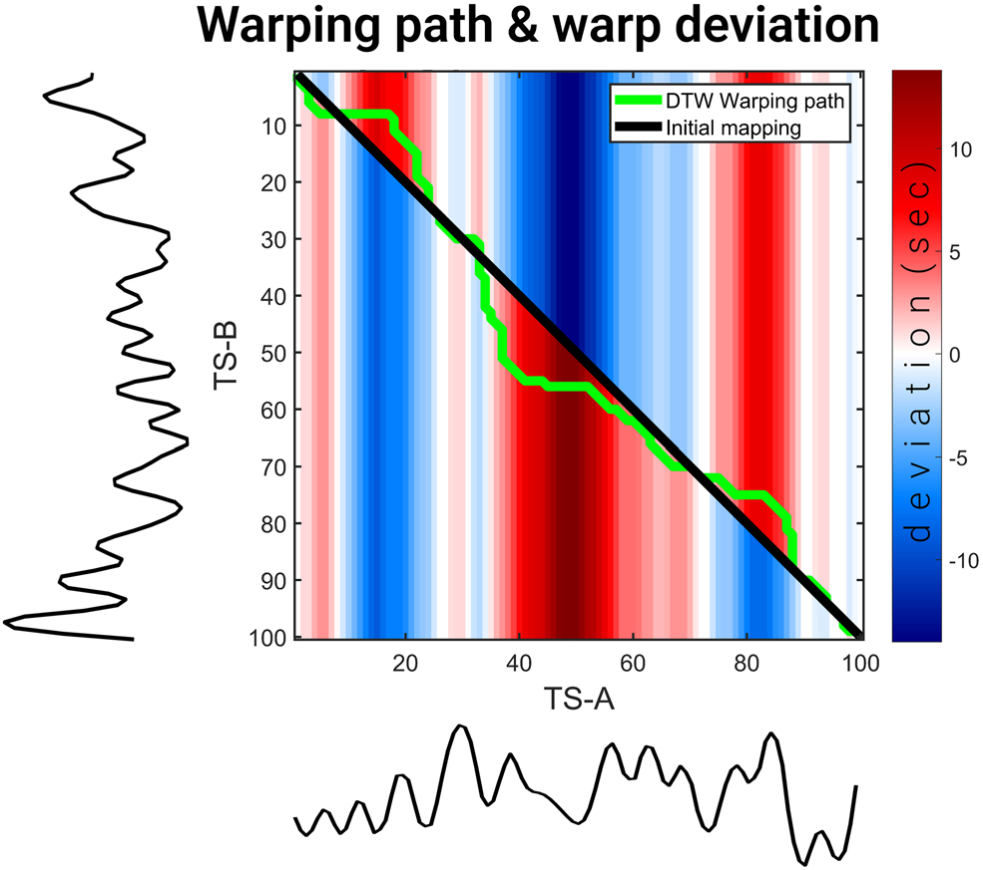
The warping path and warp deviation between two bandlimited time series signals, TS-A and TS-B, each consisting of 100 time points. The initial warping path between TS-A and TS-B is depicted by the black diagonal line, before applying DTW, while the green line showcases the warping path post-DTW alignment. To quantify the impact of DTW, we compute the warp deviation of the warping paths of TS-A and TS-B. The resulting warp deviations are presented in the heatmap: TS-B’s warp deviations occupy the lower triangle, while TS-A’s warp deviations occupy the upper triangle. Notably, a consistent color represents the warp deviation for all vertical points within each respective triangle.

The DTW algorithm aligns the time series to maximize the similarity between the signals. This means that the algorithm can map pairs of signals to various indices of each other if it optimizes the similarity between the time series pair as shown inFigure 1(B). However, the DTW algorithm also incorporates a warping window constraint. This constraint sets a limit on the extent of stretching and mapping length that can occur during the DTW alignment. For instance, without any window constraint, index 10 of time series TS-A could be mapped to index 30 of time series TS-B. Yet, when a window constraint of 10 is applied, the highest index in TS-B to which index 10 of TS-A can be mapped is 20. This constraint helps control the degree of distortion between the aligned time series while still allowing for meaningful alignments. Importantly, it should be clarified that the warping window does not dictate a predetermined level of stretching or mapping length. Instead, it represents the upper limit on the extent of stretching and mapping that is permissible within the alignment process. The effectiveness of DTW hinges on the careful choice of the window constraint. It is crucial to select a window size that strikes a balance: it should be sufficiently large to capture complex nonlinear dynamics accurately, yet not so large that it introduces less meaningful alignment of time series pairs. In this study, we used a similar approach fromMeszlényi et al. (2017)in setting the window for our DTW analysis. In fMRI, a common practice is to apply a bandpass filter with a lower limit of 0.01 Hz. This threshold corresponds to a full signal cycle of 100 seconds. Anticorrelations observed in fMRI data suggest inverse relationships between brain region activities. These anticorrelations are rather weak, transient, and less stable (Chang & Glover, 2010;Shehzad et al., 2009). Several studies have also suggested that physiological noise and global signal regression could introduce anticorrelations (Chang et al., 2009;Chang & Glover, 2010;Murphy et al., 2009;Saad et al., 2012;Shmueli et al., 2007;Wise et al., 2004). For such anticorrelations to occur, the signals might be out of phase by half a cycle, equating to a 50-second lag. Thus, analyzing the data over a 100-second window is logical, as it encompasses a full cycle of the signal, allowing for the detection of both synchronous and out-of-phase relationships (Meszlényi et al., 2017). Rather than using an approximation of 100 seconds for the DTW window, we use the activity signal spectrum to guide the selection of the window size:

$$f_{3db}=\frac{0.88}{\sqrt{N^{2}-1}}F_{s},$$ (1)

where N is the window size and$F_{s}$is the sampling frequency (1/0.72 seconds – 1.39 Hz for HCP and 1/2 seconds – 0.5 Hz for fBIRN). Because of the band pass filter applied to the data, the high-pass cutoff$f_{3db}$should be higher than 0.01 Hz which results in a minimum N value of ~123 for HCP and ~45 for fBIRN. Note 123 and 45 are the number of time points rather than seconds.

### 2.3 Warp deviation

Warp deviation quantifies the disparity between the warping paths generated by DTW for a pair of signals. In simpler terms, it measures the temporal distortion of one signal to another. The warp deviation is calculated as the temporal difference in the warping paths$i_{x}$and$i_{y}$of two time seriesxandy,respectively, at a specific time (t). The formula for the warp deviation of the time seriesxis given below:

$$WD_{x}\left(t\right)=i_{x}\left(t\right)-i_{y}\left(t\right).$$ (2)

The warp deviation of the time seriesywill simply be the negation of$WD_{x}\left(t\right)$. When$WD_{x}\left(t\right)$has positive values, it indicates a delayed response of time seriesxtoy. This is because if, at a given time (t),x, delaysy, the warping path ofx,$i_{x}\left(t\right)$will have time indexes higher than$i_{y}\left(t\right)$. This observation is significant because such delays could occur due to the stretching of time series x, causing it to lag time series y for subsequent responses. On the contrary, negative$WD_{x}\left(t\right)$values indicate that the time seriesxprogresses faster thanyin its temporal alignment. This could also suggest a cumulative shrinkage occurring in time seriesxwith respect toy.

InFigure 2, the initial mapping path is depicted by the black line, while the DTW warping path is represented by the green line. To obtain the warp deviation, we calculate the difference between the TS-A side and the TS-B side of the DTW warping path (green line inFig. 2) taking TS-A as reference for the upper triangle and negated when using TS-B as reference for the lower triangle. To visually represent the warp deviation, we utilize a colormap within the heatmap matrix depicted inFigure 2. In this matrix, the upper triangle corresponds to the warp deviation linked to TS-B, whereas the lower triangle represents the warp deviation related to TS-A. Positive warp deviation values (red stripes) show the time by which the time series delays the other while negative warp deviation values (blue stripes) show the time by which the time series progresses faster than the other.

### 2.4 Warp elasticity

Warp elasticity is designed to estimate the extent of stretching or shrinking happening between a pair of interconnected time series. We determine the degree of stretching or shrinking in a time series by calculating the temporal derivative of the warp deviation. This approach is logical because the warp deviation reflects the delay or lead of one time series to another, resulting from stretching or shrinking. As cumulative stretching leads to delay and cumulative shrinking leads to a lead in time series, taking the derivative of this delay or lead allows us to estimate the stretching and shrinking. The warp elasticity of time series x is expressed as follows:

$$WE_{x}\left(t\right)=\frac{d\left(WD_{x}\left(t\right)\right)}{dt}.$$ (3)

Since the unit of$WD_{x}\left(t\right)$is time, its temporal derivative is dimensionless. This signifies that the warp elasticity represents stretching and shrinking factors. For instance, a factor of 0.5 indicates a 50% stretching of one time series to another, while −0.5 indicates a 50% shrinkage. A factor of 0 denotes no shrinkage or stretching in one time series to the other.

We conducted a simulation study to investigate how well warp elasticity estimates the stretching and shrinking temporal adaptation of two time series to mimic the temporal adaptation of the timescale of functional connectivity among brain regions. Conceptually, one could consider that there might be adaptation in the communication scale (which for fMRI would be a combination of neural interaction and hemodynamic interactions) driven by higher-level processing networks, whereas lower-level processing networks, such as sensory networks, would have a less adaptive nature. For our toy example, we generated two signals for this purpose. The first signal, which we refer to as TS-A, consisted of a random signal with a length of 210 time points, sampled at a rate of 1 second. To analyze the adaptation of processing speed, we divided this TS-A signal into seven equal segments, each containing 30 time points, collectively covering the 210-timepoint duration. To create the second signal, denoted as TS-B, we resampled each of these seven segments to specific lengths: 20, 40, 20, 30, 40, 20, and 40 time points, respectively. The sampling factor that yields the new lengths is computed by dividing the initial timepoint length by the new length of timepoints. Thus, the sampling rates for these seven segments are 0.67, 1.33, 0.67, 1, 1.33, 0.67, and 1.33, respectively. These sampling factors represent the stretching and shrinking of TS-B. Subsequently, we concatenated these resampled segments to form the TS-B time series. Random noise was introduced to time series data sets TS-A and TS-B, followed by band limiting both TS-A and TS-B to a frequency range of 0.01 to 0.15 Hz, aligning with the parameters utilized in our fMRI analysis. InFigure 1(C), we show TS-A and TS-B while highlighting the corresponding segments of resampling occurring in TS-B using black bars. InFigure 1(D), the warp deviation of TS-A is depicted. It is observed that when the sampling factor is lesser than 1, indicating shrinking in TS-B and relative stretching in TS-A, there is a consistent rise in the warp deviation of TS-A. Conversely, when the sampling factor is greater than 1, denoting a relative shrinkage in TS-A, there is a continual decrease in the warp deviation. Moreover, when the sampling rate is 1, indicating no change in size, a constant value is maintained within that segment. As anticipated, the stretching and shrinking patterns in the segments are reflected in the rate of change of the warp deviation.

InFigure 1(E), the warp elasticity of TS-A alongside the ground truth elasticity is illustrated. In our ground truth simulation, we generated 1000 random time series pairs with the same stretch and shrink structure as described earlier. For each pair, we computed the warp elasticity measure independently. Subsequently, we averaged the warp elasticity values across all these random samples. Finally, we compared these average warp elasticity values with the ground truth, allowing us to validate the accuracy of our methodology across various random scenarios. The ground truth elasticity is calculated by determining how many time points a segment in one time series has been stretched or shrunk and dividing it by the total length of the time series’ time points in that segment. For instance, in the initial segment of our example where TS-B was sampled with a factor of 0.67, it means TS-B has 20 time points while TS-A has 30 time points. Considering TS-A, there is a relative stretch of 10 time points compared with TS-B. Hence, the actual stretch factor for TS-A in that segment is +0.33 [10/30]. Positive values denote stretching, and negative values indicate shrinking. InFigure 1(E), the ground truth elasticity factor of TS-A is represented by a black dashed line, and the estimated warp elasticity is shown in red. As depicted inFigure 1(E), the correlation coefficient between the ground truth elasticity and the warp elasticity measure is 0.88, indicating a strong correlation between the ground truth and the warp elasticity measure. This simulation example effectively demonstrates how our method, the warp elasticity, can reveal time-varying stretching and shrinking behaviors in signals as they adapt to one another. This sheds light on the intricate dynamics of signal alignment, showcasing the potential of our approach to understanding the nuanced interactions between signals.

Since the warp elasticity estimates are functions of sampling time, we accommodate for changes in warp elasticity relative to sampling time by expressing it in seconds. For instance, consider a 30-second segment with a stretching factor of 0.5. This means that for every second, the time series is stretched by 0.5 seconds, leading to a total stretching of 15 seconds over the 30-second segment, regardless of the sampling rate. Thus, expressing warp elasticity values in seconds ensures consistency, facilitating comparisons across datasets with different sampling rates. This methodology becomes particularly relevant in our study, where we are analyzing datasets with different temporal resolutions. For example, the fBRIN dataset has a TR of 2 seconds, whereas the HCP dataset has a TR of 0.72 seconds. By standardizing warp elasticity in relation to sampling time, we aim to make the results from these different datasets comparable and consistent. This approach will be a key aspect of our subsequent analyses in this study as it also provides us with a way to view the estimations in terms of seconds instead of constants.

### 2.5 fMRI data

This study received approval from an ethics board, and all participants provided consent by signing a form approved by the institutional review board (IRB).

Our first dataset included resting-state fMRI data collected from 827 subjects via the Human Connectome Project (HCP) database (Van Essen et al., 2012,^2013^). Specifically, we analyzed second-session scans acquired using a Siemens Skyra 3T scanner with a multiband accelerated, gradient-echo echo-planar imaging (EPI) sequence. The scanning parameters were set to a repetition time (TR) of 0.72 seconds, 72 slices, an echo time (TE) of 58 ms, and a flip angle of 90°. A voxel size of 2 × 2 × 2 mm was used to acquire 1200 time points of fMRI data for each subject. The use of the HCP dataset in our study served two primary objectives. Foremost, it established the foundational basis for evaluating the noise sensitivity of our metric. The rationale behind the selection of the HCP dataset emanated from its notable high-quality and meticulous preprocessing standards, coupled with an extensive participant pool that can assess the robustness of our metric evaluation. Moreover, the dataset’s widespread adoption within the neuroscience community facilitated facile cross-study result comparisons, thus accentuating its suitability for our research.

Secondly, we used data from SZ patients and controls collected via the Function Biomedical Informatics Research Network (fBIRN) study. For participants to be included, they needed to meet specific criteria: their head motion during scans had to be equal to or less than 3° and 3 mm, and their functional data should demonstrate successful normalization by aligning individual brain masks with a group mask. The scans were collected at a repetition time (TR) of 2 seconds. These conditions led to a total of 160 HCs with an average age of 37.04 ± 10.86 years, ranging from 19 to 59 years. Among these, 45 were female and 115 were male. Additionally, there were 151 patients diagnosed with SZ, with an average age of 38.77 ± 11.63 years, ranging from 18 to 62 years. In this group, 36 were female and 115 were male. The HCs and SZs were meticulously matched in terms of age, gender distribution, and mean framewise displacement during scans (age: p = 0.1758; gender: p = 0.3912; mean framewise displacement: p = 0.9657). Notably, the HCs had no history of past or current psychiatric disorders based on the Structural Clinical Interview for Diagnostic and Statistical Manual of Mental Disorders assessment, nor did they have first-degree relatives diagnosed with Axis-I psychotic disorders. It is important to highlight that the SZs, while diagnosed with schizophrenia, were in a clinically stable condition during the time of their scans. In our study, we employed the fBIRN dataset for conducting group comparisons, aiming to assess the capability of our method in identifying significant differences between the groups, specifically SZ and HC.

### 2.6 fMRI processing

Functional magnetic resonance imaging (fMRI) data require extensive preprocessing to correct for various sources of noise and artifacts before analysis. The preprocessing steps commonly applied in fMRI studies include slice timing correction, realignment, spatial normalization, and spatial smoothing (Esteban et al., 2019;Penny et al., 2011;Turner et al., 1998). Following preprocessing, we implemented the NeuroMark pipeline, a fully automated spatially constrained ICA on the preprocessed fMRI data (Du et al., 2020). Using the neuromark_fMRI_1.0 template, we generated 53 intrinsic connectivity networks (ICNs) for each subject. These ICNs are grouped into brain domains including Subcortical (SC), Auditory (Aud), Sensorimotor (SM), Visual (Vis), Cognitive Control (CC), Default Mode (DM), and Cerebellum (Cb). To further enhance the quality of these ICNs, we applied detrending and despiking techniques to remove drifts, abrupt fluctuations, and large artifacts that may not have been removed by the initial preprocessing steps. These techniques helped improve the accuracy and reliability of subsequent analyses.

To optimize data quality, the time series of intrinsic connectivity networks (ICNs) were bandpass filtered in the range of 0.01 to 0.15 Hz, a standard frequency range in fMRI research that is relevant for identifying brain domain BOLD signals (Braun et al., 2012;Yaesoubi et al., 2015). An infinite impulse response (IIR) filter was designed using the butter function in MATLAB applied via the filtfilt function to ensure zero phase shifts and preserve phase information, which can be nonlinearly altered by IIR filters (Caballero-Gaudes & Reynolds, 2017;Oppenheim, 1999;Proakis, 2007). Finally, z-scoring was performed on the ICNs.

### 2.7 Analysis

In our research, we conducted a series of rigorous analyses to establish the credibility of warp elasticity as a robust metric. Our primary objectives encompass demonstrating the metric’s replicability and robustness, while also showcasing the novel insights it offers for interpreting fMRI data. Through these analyses, we affirm the metric’s reliability as a viable metric for neuroimaging analysis. Furthermore, we showcase how warp elasticity reveals promising patterns in fMRI, thus contributing to an enhanced comprehension of neural dynamics.

In our study, we utilize k-means clustering to group the data derived from warp elasticity estimations. This technique helps us identify recurring temporal patterns in the warp elasticity of component pairs across various subjects, following the methodology described byAllen et al. (2014). These identified patterns are referred to as “functional connectivity states” in FC analysis, but in this study, we refer to them as “warp elasticity (WE) states.” By analyzing these states, we can track the transitions between them and quantify the duration subjects spend in each state. This is particularly insightful as it may highlight differences between healthy individuals and patients with brain disorders (Du et al., 2018;Faghiri et al., 2023;Fu et al., 2018;Kazemivash & Calhoun, 2022;Kazemivash et al., 2023;Kim et al., 2017;Sang et al., 2023;Sun et al., 2021). This technique is useful for checking the consistency of trFNC measures and comparing different trFNC methods, ensuring reliable and robust results in neuroscience research (Abrol & Calhoun, 2022;Abrol et al., 2017;Faghiri et al., 2022;Motlaghian et al., 2023;Romanello et al., 2022;Wiafe et al., 2023). The WE states represent global recurring stretching and shrinking patterns between brain network pairs. In essence, each state encapsulates a recurring stretching or shrinking pattern exhibited by brain region pairs throughout the fMRI scan. These patterns occur at different times within the scan, offering insight into the global stretching and shrinking recurring dynamics across the brain.

#### 2.1.1 Comparison with existing trFNC measures

Our study is centered on investigating the stretching and shrinking parameter, which is not addressed by existing trFNC methods such as SWPC or PC. However, we offer a simulation to compare warp elasticity with these trFNC measures across pairs of time series exhibiting stretching and shrinking. This aims to emphasize the differences between warp elasticity and trFNC measures, specifically SWPC and PS. SWPC is calculated using various window sizes—specifically 15, 45, 75, 105, and 135 seconds—to comprehensively assess its capability to detect or account for stretching and shrinking across different coupling timescales. The SWPC of a time series pair, denoted asx(t)andy(t), for a selected window,Δ, is expressed as follows:

$$SWPC_{x,y}{\left(t\right)}_{\Delta }=\sum_{\Delta =t-\Delta /2}^{t+\Delta /2}\frac{\left(x{\left(t\right)}_{\Delta }-\bar{x}{\left(t\right)}_{\Delta }\right)\left(y{\left(t\right)}_{\Delta }-\bar{y}{\left(t\right)}_{\Delta }\right)}{\sigma {\left(t\right)}_{\left(x,\Delta \right)}.\sigma {\left(t\right)}_{\left(y,\Delta \right)}}.$$

PS is computed by determining the difference in the estimated instantaneous phase of a signal pair using the Hilbert transform. To ensure comparability with SWPC, a function is applied to constrain the phase difference results between −1 and 1. The cosine function is useful for this purpose as it preserves anticorrelations, avoids phase unwrapping, and considers phase ambiguity (Honari et al., 2021). Given a time series pair, denoted asx(t)andy(t), along with their corresponding estimated instantaneous phases represented as$\varphi_{x}\left(t\right)$and$\varphi_{y}\left(t\right)$using the Hilbert transform, the phase synchrony betweenx(t)andy(t)is expressed as follows:

$$PS_{xy}\left(t\right)=\cos\left(\varphi_{x}\left(t\right)-\varphi_{y}\left(t\right)\right).$$

Given that PS is sensitive to the selection of an appropriate filter bandwidth, as per the Bedrosian theorem (Bedrosian, 1962;Xu & Yan, 2006), we conduct PS for various bandwidths: 0.01–0.04 Hz, 0.03–0.07 Hz, 0.05–0.09 Hz, 0.07–0.11 Hz, and 0.09–0.13 Hz using a Butterworth filter.

Similar to the simulation conducted in the warp elasticity section and as illustrated inFigure 1, we generate 1000 pairs of time series, each comprising 400 timesteps, with predetermined relationships. In the initial half of each pair, the time series are Gaussian random, indicating no relationship across all samples. However, for the latter half of the signal pairs, we generate 50-second segments of random signals for TS-A and subsequently resample them at varying sampling rates to create TS-B, thereby inducing time-varying stretching and shrinking effects in their couplings. The resampling rates applied to the second half to create TS-B are 0.6, 1.4, 0.6, and 1.4 as illustrated inFigure 3(A).

**Fig. 3.**
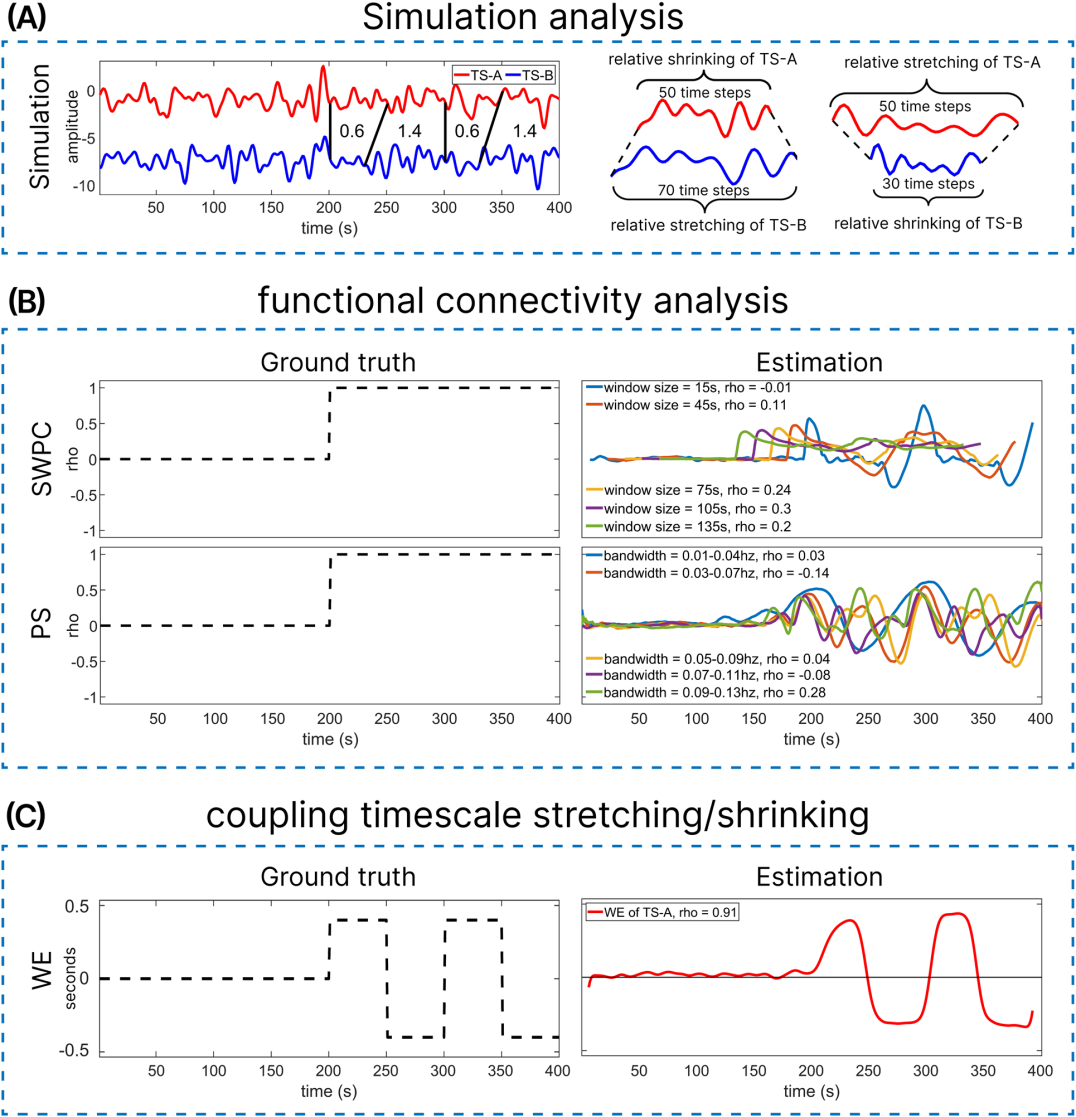
The performance of traditional functional connectivity measures, including sliding window Pearson correlation (SWPC) and phase synchrony (PS), amidst varying timescales of coupled time series. (A) Simulations involving 400-point time series pairs. Each pair begins with independent and identically distributed (IID) data for the first half of both time series, denoted as TS-A and TS-B. For the latter half, time-varying coupling timescale stretching and shrinking are introduced by resampling 50-second segments of TS-A to create TS-B. (B) The estimation of functional connectivity analysis through the application of SWPC with different window sizes and PS with varying bandwidths. These estimations are compared against their respective ground truths for assessment. The time-varying coupling timescale stretching/shrinking is estimated utilizing warp elasticity (WE), as depicted in (C), and then compared against its ground truth for validation.

In this simulation, for the initial half of the time series pairs, we anticipate that both SWPC and PS will estimate the ground truth relationship of zero. This expectation arises from the fact that these signals are generated using a univariate Gaussian distribution independently. Consequently, we expect warp elasticity to exhibit no stretching or shrinking effects, resulting in a value of 0 seconds. For the second half, we anticipate that both SWPC and PS will exhibit high correlation and high synchrony, respectively, close to a value of 1 for both measures. This expectation arises from the fact that the segments are perfectly synced and correlated, with the only variation being due to different temporal resolutions induced by the resampling rates. Warp elasticity is anticipated to quantify the amount of stretching and shrinking in seconds, aligning with the resampling factors as computed in the warp elasticity section. The ground truth for SWPC and PS is depicted inFigure 3(B), while the ground truth for warp elasticity is illustrated inFigure 3(C). The difference in ground truths for trFNC measures and warp elasticity underscores the differences between these measures. trFNC evaluates the time-resolved connectivity between a pair of signals, whereas warp elasticity measures the temporal distortions specifically, stretching and shrinking, of the couplings between a pair of signals.

#### 2.1.2 Noise sensitivity

We examined the impact of noise on warp elasticity by introducing varying levels of noise to the fMRI signals. This approach enabled us to establish distinct signal-to-noise ratios (SNRs) for our data during the evaluation process. Treating the HCP fMRI data as the pure signal, we systematically added noise of different intensities to achieve specific SNR values. We are interested in observing the sensitivity of warp elasticity in a specific range of SNRs that is associated with fMRI. Several studies have reported the SNR range of fMRI data (Hahn & Rowe, 2012;Hughes & Beer, 2012;Särkkä et al., 2012;Welvaert & Rosseel, 2013).Welvaert and Rosseel (2013)provided a concise overview of multiple research papers that discussed the SNR in fMRI and revealed that the temporal SNR (tSNR) of fMRI is within the range of 1.07 and 84.54 with a mean of 12.98. tSNR is a term used for computing the SNR for fMRI time. The typical way of estimating tSNR involves dividing the mean value of the fMRI time by the variance of the noise time series (Nan & Nowak, 1999;Triantafyllou et al., 2005;Welvaert & Rosseel, 2013). For this reason, our noise sensitivity test does not include the z-scoring step in our fMRI postprocessing.

$$tSNR=\frac{\bar{S}}{\sigma_{N}},$$ (2)

where$\bar{S}$is the mean of the fMRI time series and$\sigma_{N}$is the variance of the noise time series.

To test how sensitive our metric is to different levels of noise, we introduce noise signals at different variance levels to the fMRI time series, aligning these variances with specific tSNR values. We do this by generating 100 different noise signals at various temporal signal-to-noise ratio (tSNR) levels for each brain network time series independently. These tSNR levels were randomly sampled from a Gaussian distribution with a mean of zero and a standard deviation corresponding to the targeted tSNR value. It is important to note that the noise signals added to each brain network were individually created, ensuring that the variability in noise across networks was captured in our analysis. These tSNR levels ranged from 100 to 0.01, with a specific focus on values within the range of 84.54 to 1.07. For each tSNR value, we followedEquation (2)to create 100 sets of noise samples. These noise samples were then added to the original fMRI data, resulting in the generation of 100 distinct noisy fMRI signals for each tSNR and each brain network independently. This entire process was repeated for all pairs of brain networks. We calculated correlation coefficients between the warp elasticity of noisy fMRI signals and the warp elasticity of the actual fMRI signals at each SNR value. Our goal is to examine the correlation between each noisy fMRI time series and the original, noise-free fMRI time series. We anticipate observing a high correlation, even for low tSNR values, demonstrating that our warp elasticity method is less sensitive to noise.

We conducted a noise sensitivity assessment on simulated data, mirroring the tSNR range utilized in the fMRI noise sensitivity analysis. We created 100 random pairs of time series, each pair with their corresponding ground truth. Subsequently, we generated 100 random pairs of noise signals from a Gaussian distribution with a mean of zero and a standard deviation aligned with the targeted tSNR value. These noise signals were added to the signal pairs representing the ground truth stretching/shrinking. To evaluate the effect of noise on our estimates, we compared the estimations of WE between the signals with varying tSNRs and their respective ground truths using correlation analysis.

#### 2.1.3 Bootstrapping

In this analysis, our primary objective is to demonstrate the robustness of our proposed metric, warp elasticity. To achieve this, we leverage the HCP dataset due to its higher participant pool, which offers a higher subject count compared with the fBIRN dataset. Our approach involves randomly selecting four sets of 200 subjects from the HCP dataset. To assess the robustness of the warp elasticity across different subject compositions, we implement our novel methodology on the four selected subject sets separately to obtain the estimated measures for both sets. We scale the warp elasticity values by a factor of 0.72 since that is the TR of the HCP dataset.

Subsequently, we employ the k-means clustering algorithm on the warp elasticity measure, employing the city-block distance function, to cluster the time-resolved vectors resulting from our estimations. This analysis allows us to explore the consistent patterns of connectivity across different subject subsets. We selected a cluster number of 3 for this analysis based on the elbow criterion, by taking the ratio of the within-cluster sum of squared distances (WSS) to the between-cluster sum of squared distances (BSS) (seeSupplementary Materialfor elbow plot).

#### 2.1.4 Group comparison

We employed the fBIRN dataset to assess the effectiveness of warp elasticity in discerning group disparities, specifically between individuals with schizophrenia and a healthy control group. Our approach involved applying warp elasticity across the entire fBIRN dataset. We scale the warp elasticity values by a factor of 2 since that is the TR of the fBIRN dataset. Subsequently, we employed k-means clustering with a city-block distance metric to obtain recurring warp elasticity states (Allen et al., 2014). We chose 3 clusters based on the elbow criterion, by taking the ratio of WSS to BSS (seeSupplementary Materialfor elbow plot). The k-means clustering is performed on the full fBIRN dataset including HC and SZ groups, and subsequently separated the clusters based on group membership (HC and SZ), allowing for a direct comparison between the two groups. After clustering, we visualized the obtained clusters. In addition, to quantify the changes over time, we computed group-specific mean dwell times, percentage occupancy (fraction rate) for each state, and a transition matrix. “Mean dwell time” is defined as the average duration that a subject remains within a specific cluster after entering it; “fraction rate” refers to the average portion of time a subject spends in a particular cluster; “transition matrix” is a matrix that quantifies the probability of a subject transitioning from one cluster state to another (Allen et al., 2014;Iraji et al., 2020). In this study, we refer to the metrics of mean dwell time, fraction rate, and the transition matrix collectively as WE state dynamics.

In our analysis comparing individuals with SZ and HC, we used the two-sample*t*-test to examine differences in the WE state dynamics. The*t*-test is appropriate for comparing two independent groups and assumes normally distributed data with equal variances. To address the risk of Type I errors due to multiple comparisons, we applied the False Discovery Rate (FDR) correction, specifically the Benjamini-Hochberg procedure. This approach adjusts p-values to account for the proportion of expected false positives. Our significance threshold was set at an FDR-adjusted p-value of less than 0.05, following common conventions to control for multiple comparisons. This threshold was chosen to ensure that our findings are both statistically robust and meaningful, reflecting true differences in the WE state dynamics between the SZ and HC groups.

## 3 Results

### 3.1 Comparison with existing trFNC measures

Figure 3demonstrates the performance of two trFNC measures, sliding window Pearson correlation and phase synchrony, in the context of varying coupling timescales. InFigure 3(B), we present the estimations of SWPC and PS alongside their respective ground truths. The estimation with each window size for SWPC and each bandwidth for PS is compared with the ground truths using Pearson correlations. These correlation values are depicted in the estimation section ofFigure 3(B). The initial half of SWPC and PS estimations across all 1000 samples effectively capture the ground truth of zero synchrony and correlation, as expected.

The second half demonstrates poor performance in estimating the ground truth using both SWPC and PS methods. The reason for this poor performance is the varying temporal coupling timescales. Specifically, in SWPC, smaller window sizes exhibit spikes at the points where stretching or shrinking begins to occur between TS-A and TS-B, notably for the 15 seconds window size and around time points 200, 300, and 400 seconds. Higher window sizes also capture these transient couplings, but due to their longer window lengths, these transients are estimated slightly early. Similarly, this effect is observed in the estimation of PS for all bandwidths. It is worth noting that, as shown inFigure 3(A), time points 200, 300, and 400 represent time points where stretching or shrinking initiates or ends between TS-A and TS-B. Consequently, points around those instances show no significant stretching or shrinking between TS-A and TS-B. It is evident that in the second half of TS-A and TS-B, negative phase synchronization and negative correlations occur for PS and SWPC, respectively. This phenomenon arises because of the stretching and shrinking effects present in the data.

Warp elasticity effectively estimates no stretching or shrinking for the initial half of the signal pairs, TS-A and TS-B across all 1000 samples. In the second half of TS-A and TS-B, warp elasticity accurately estimates the coupled stretching and shrinking timescale, demonstrating a correlation of 0.91 with the ground truth, as depicted inFigure 3(C).

### 3.2 Warp elasticity noise sensitivity

**Fig. 4.**
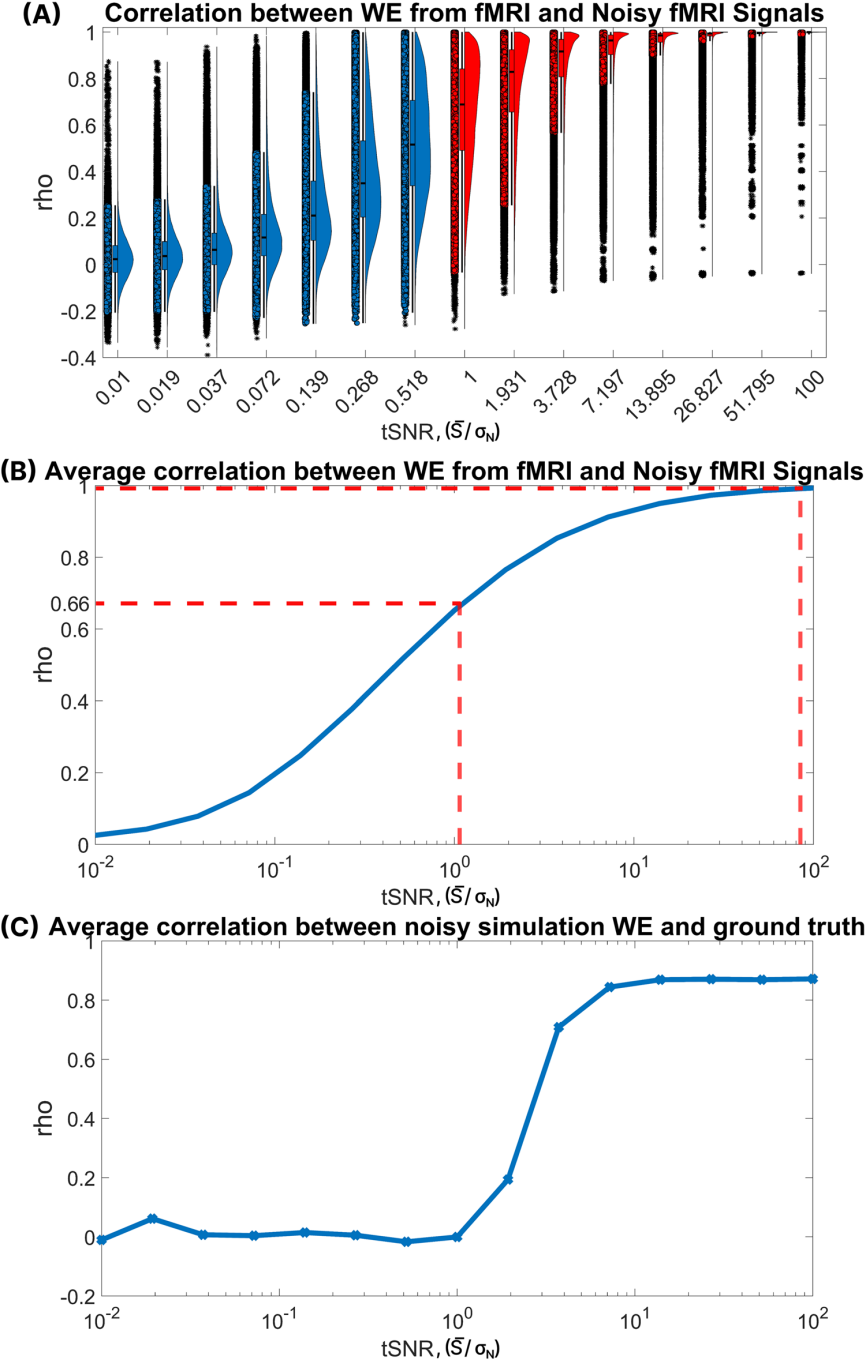
An analysis of correlation coefficients utilizing various temporal signal-to-noise ratio (tSNR) values, spanning from 0.01 to 100 on a logarithmic scale. This encompasses 15 tSNR values logarithmically distributed. Correlations were computed between warp elasticity of noisy HCP fMRI time series and actual HCP fMRI time series (A) and (B), and between warp elasticity of noisy simulated data and the ground truth (C). For each tSNR value, 100 sets of noisy signals were generated (B). For (A) and (B), 100 sets of noisy signals were generated for each pair of functional networks. The distribution of correlation coefficients is visualized for all 100 samples of functional network pairs. The distributions relevant to fMRI tSNR values (ranging from 1.07 to 84.54) are highlighted in red in (A). The average correlation coefficient for all random samples and component pairs is depicted in (B) for each tSNR value. Consequently, each tSNR value is associated with a single correlation coefficient value. Red dotted lines on the plot pinpoint the tSNR values that align with the tSNR of typical fMRI data (between 1.07 and 84.54). Similarly, (C) depicts the average correlation coefficients across all random samples for each tSNR value between the simulated data and the ground truth.

We first examined warp elasticity sensitivity to noise using the HCP dataset. We generated 100 noisy fMRI signals at tSNR levels ranging from 100 to 0.01. Correlation coefficients between warp elasticity of noisy and actual fMRI signals were calculated, revealing noise impact on warp elasticity. The results are illustrated inFigure 4, which displays the correlations between the noisy fMRI signals and the actual fMRI signals using various visual representations. InFigure 4(A), the histograms depict the distribution of correlation coefficients across all component pairs and samples for each specific tSNR value. Each tSNR value is represented by a histogram, with the red histograms highlighting the range most relevant to fMRI (1.07 to 84.54), as previously mentioned.Figure 4(A)*also*showcases box plots and scatter plots, offering an alternative visualization of the information presented in the raincloud plot. These provide a clearer insight into the presence of outliers within the correlation coefficient distributions compared with the histograms.

The relevant fMRI tSNR histograms withinFigure 4(A)(depicted by the red histograms) reveal an interesting observation: the distribution of correlation coefficients is primarily clustered at 1, spanning from tSNR values of 100 down to approximately 7. This indicates a strong positive correlation between the variables. For tSNR values ranging from 3.7 to 1, the correlation coefficients vary from 1 to 0. Most of the coefficients are close to 1, signifying high positive correlations, with only a minor tail extending toward correlation coefficients close to 0. Also, certain samples or network pairs display relatively low correlation values, as evidenced by the box and scatter plots inFigure 4(A). This important visual cue emphasizes that while our method demonstrates reduced sensitivity to noise, a subset of samples/network pairs still exhibit correlations that diverge from the typical trend. The significance of this variation, however, is very small and can be safely disregarded. Nevertheless, we believe it is important to present these findings to provide a comprehensive perspective on the outcomes of our study.

Figure 4(B)provides an overview of the average correlation between noisy signals and the actual fMRI data, encompassing various tSNR values. The red dashed lines emphasize the relevant tSNR range for fMRI (1.07 to 84.54). The plot demonstrates strong associations (correlation coefficients ranging from 1 to ~0.66) between noisy fMRI and actual fMRI signals for the relevant fMRI tSNR range. For an evaluation of the global performance of warp elasticity, averaging the correlation coefficients across all samples and network pairs provides the most accurate assessment of its noise sensitivity. This goes on to show that the proposed warp elasticity method exhibits remarkable resilience in the presence of noise particularly in fMRI data.

Furthermore, we examined the noise sensitivity of warp elasticity with ground truth elasticity from simulated data.Figure 4(C)illustrates the average correlation between warp elasticity of simulated data with varying tSNR values and the ground truth stretching and shrinking factors. Warp elasticity exhibits correlation coefficients exceeding ~0.71 for tSNR values ranging from 100 to 3.72. However, for tSNR values below ~1.93, the reliability of warp elasticity diminishes in the simulation noise sensitivity analysis.

### 3.3 Bootstrapping

**Fig. 5.**
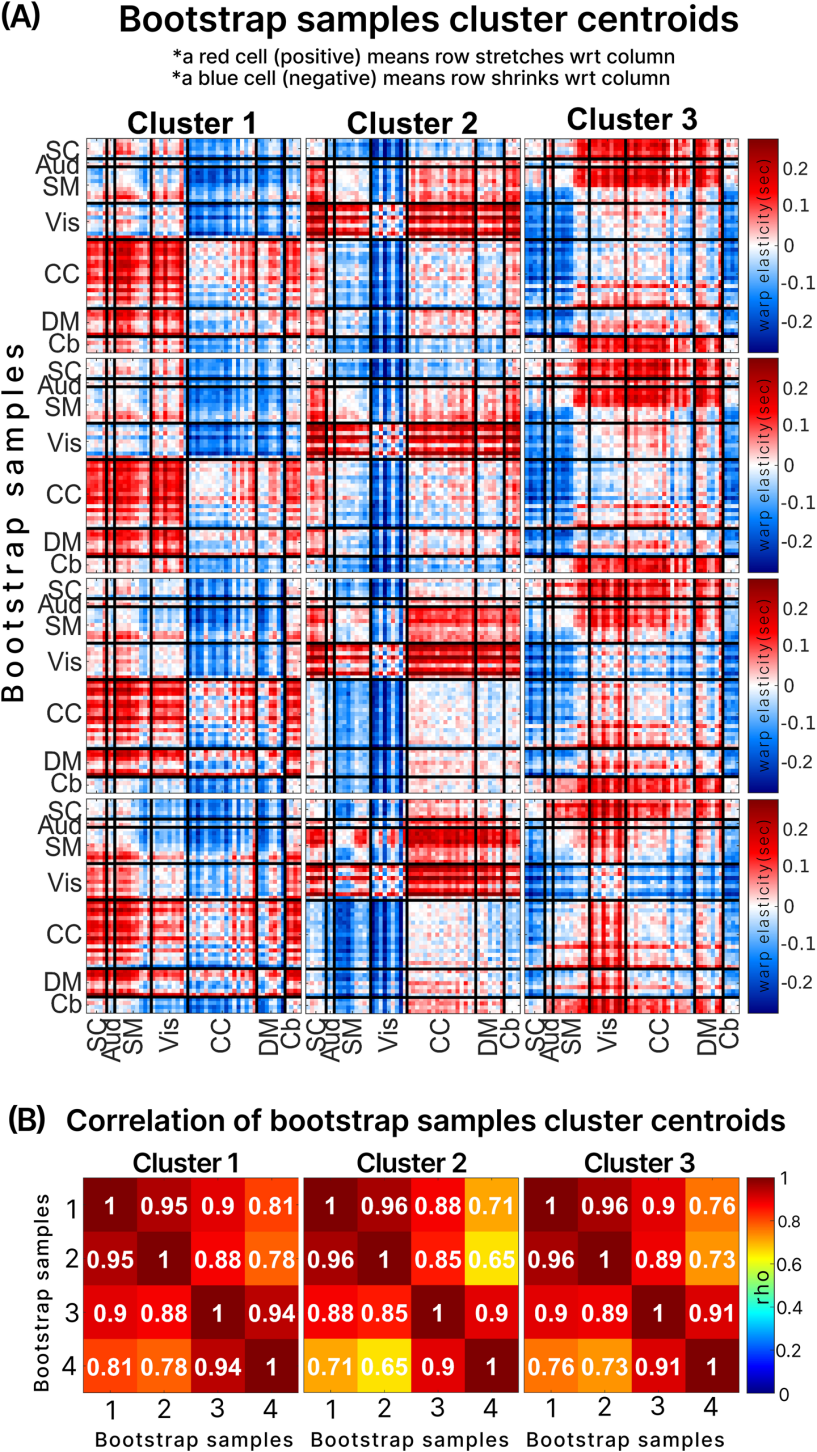
The replicability of warp elasticity through a bootstrapping analysis involving four randomly selected samples, each consisting of 200 subjects from the extensive HCP dataset. Within each of these four subset samples, we applied the warp elasticity method and subsequently performed k-means clustering with 3 clusters. (A) The resulting cluster centroids for each sample are visually presented, with the individual samples displayed in rows and the corresponding cluster centroids in columns. These cluster centroids are characterized in terms of their brain domains. (B) Cross-correlation between cluster centroids across all bootstrap samples for each cluster number.

Figure 5illustrates the robustness of the warp elasticity method through a bootstrap replicability test. We conducted this test by randomly selecting four sets of 200 subjects from the HCP dataset and independently applying the warp elasticity method to each set. Subsequently, we utilized k-means clustering with three clusters to group the estimated warp elasticity and displayed their cluster centroids inFigure 5(A). The rows inFigure 5(A)correspond to the four bootstrap samples, while the columns represent the three clusters for each bootstrap sample. The results show a high level of similarity between clusters of the bootstrap samples. The correlation matrix of all pairs of bootstrap samples for each cluster is shown inFigure 5(B). InFigure 5(B), it is evident that sample 4 exhibits the lowest correlation coefficients compared with all other samples in all clusters. The minimum coefficient, 0.65, is observed between samples 4 and 2 in cluster 2. However, samples 1, 2, and 3 showcase correlation coefficients above 0.85.

### 3.4 Group comparison

**Fig. 6.**
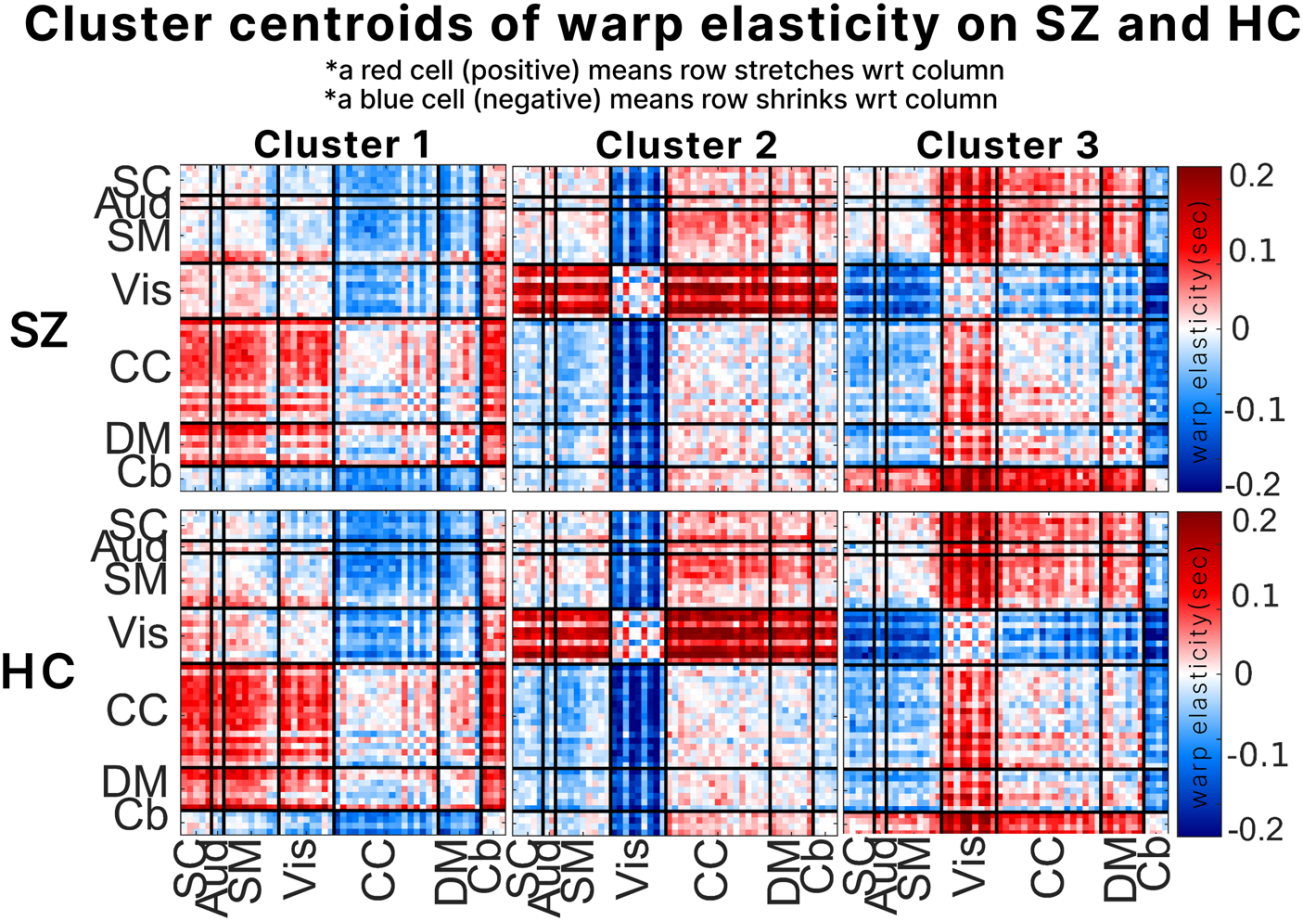
The cluster centroids derived from the fBIRN dataset for both the schizophrenia and healthy control groups, following the computation of the warp elasticity method. In the first row, we present the results for the schizophrenia group, while the second row displays the outcomes for the healthy control group. Positive values represent the stretching factor and negative values represent the shrinking factor of row networks with respect to column networks.

We conducted a group comparison analysis using the fBIRN dataset to assess the effectiveness of our method in capturing differences between SZ and HC groups. We employed the warp elasticity method on the entire dataset comprising 311 subjects. These measures were subsequently summarized using k-means clustering, resulting in WE states. We isolated the WE states associated with the SZ and HC groups from the full dataset.Figure 6displays the cluster centroids of these WE states for both SZ and HC. The warp elasticity values represent the estimated stretching and shrinking between brain networks. Positive values indicate stretching, while negative values indicate shrinking. InFigure 6, the pairwise warp elasticity between brain networks forms a nonsymmetric matrix. However, it is crucial to interpret these results following the conventional approach, reading them row by column. This is a result of the phenomenon of stretching and shrinking discussed earlier; when one network stretches against another, the latter shrinks by the same magnitude. Hence, the upper and lower triangles of the matrix are essentially mirror images of one another.

For instance, in cluster 2, the visual networks (Vis) in the row display positive values of about +0.2 in the lower triangle with respect to subcortical (SC), auditory (Aud), and sensory-motor (SM) networks in the column. This indicates that Vis networks stretch approximately 20% with respect to SC, Aud, and SM networks. Conversely, if SC, Aud, and SM networks are to be considered, they should be read from the rows, and Vis from the columns. In this case, negative values of about −0.2 in the upper triangle signify approximately 20% shrinking of SC, Aud, and SM networks with respect to Vis. These observations are consistent with the analogy of stretching and shrinking, indicating that stretching in one network corresponds to relative shrinkage of the same amount in the other network.

**Fig. 7.**
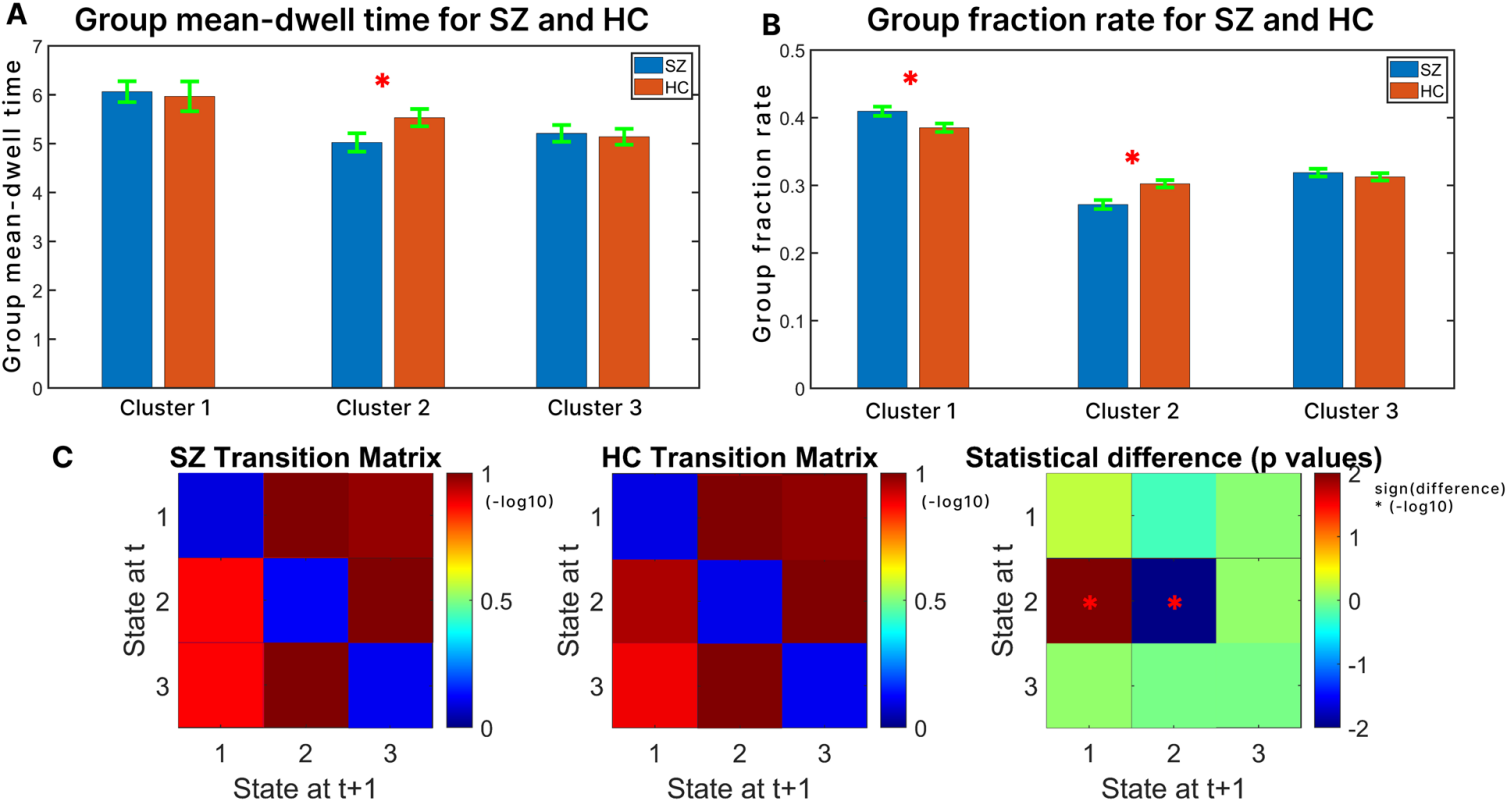
An overview of the mean dwell time, fraction rate, and transition matrices for both schizophrenia (SZ) and healthy control (HC) groups, following the computation of warp elasticity and the subsequent creation of three clusters. (A) The figure showcases the group mean dwell times for both SZ and HC groups across each cluster. The green error bars represent the standard errors associated with the mean dwell times for each group across clusters. (B) Similarly, the group fraction rates for the SZ and HC groups within each cluster are depicted, with corresponding green error bars indicating the standard errors for the fraction rates. (C) In this section, the transition matrix is presented for both the SZ and HC groups, alongside the false discovery rate (FDR) corrected p-values obtained from a two-sample*t*-test between the warp elasticity of SZ and HC cluster centroids. The scale of the transition matrices is expressed in terms of$-{\text{log}}_{10}10$. The statistical differences (p-values) are represented as the sign of the difference between the SZ and HC transition matrices, multiplied by the$-{\text{log}}_{10}10$of the FDR corrected p-values. Asterisks (*) are used to indicate significant differences between SZ and HC, as determined by the two-sample*t*-test.

To analyze the group differences between the HC and SZ groups using warp elasticity, we assessed the mean dwell times, fraction rates, and transition matrices for these two groups based on the identified WE states.Figure 7(A)displays the group (average) mean dwell times for both SZ and HC. Notably, an asterisk (*) accompanying the group mean dwell times for cluster 2 highlights a statistically significant difference between the SZ and HC groups using a false discovery rate (FDR) corrected two-sample*t*-test. The green error bars in the figure represent the standard error associated with the group mean dwell times. Similarly, inFigure 7(B), the group fraction rates for SZ and HC are illustrated with clusters 1 and 2 exhibiting significant differences.Figure 7(C)provides insight into the transition matrices by showing the$-log_{10}10$transformation of these matrices for SZ and HC in the first two matrices. In the third matrix, the sign of the difference in transition matrices between SZ and HC is multiplied by the$-log_{10}10$transformation of the false discovery rate (FDR) corrected p-values of the transition matrices of SZ and HC. The analysis highlights statistically significant differences in the transitions from WE state 2 to state 2 (noted as 2→2), and from state 2 to state 1 (2→1), as indicated by the corresponding cells (2,2) and (2,1) in the matrices ofFigure 7(C). These transition cells reveal significant differences between subjects with SZ and HC. Specifically, the differences are noted in the probability of subjects remaining in WE state 2 (for 2→2 transition) and the probability of subjects transitioning from state 2 to state 1 (for 2→1 transition). This suggests a distinct pattern in the WE state transitions for individuals with schizophrenia compared with healthy individuals.

## 4 Discussion

The findings presented regarding the warp elasticity method underscore the reliability of DTW as a robust measure for estimating the dynamics of connectivity timescales by accommodating the temporal stretching and shrinking of brain region couplings. In our study, we adhered to standard preprocessing procedures for fMRI data, which involved utilizing ICA to extract intrinsic brain networks. Additionally, we implemented rigorous quality control steps, including detrending, despiking, filtering, and z-scoring. Subsequently, we applied DTW to pairs of brain networks and quantified the warp elasticity.

Our investigation focused on discerning consistent patterns of stretching and shrinking among brain network couplings, as captured through cluster centroids while investigating the ability of warp elasticity in discerning group differences specifically SZ and HC groups. This approach provided valuable insights into how different brain regions adapt to each other over time. Importantly, the limitation associated with SWPC, which employs fixed windows for computations, was effectively mitigated by the concept of warp elasticity.Figure 1, our illustrative example, demonstrates that inFigure 1(C) and (E), each segment (window) from the ground truth aligns with one another, even when these segments undergo shifts. This illustrates that warp elasticity effectively captures the stretching and shrinking of time series couplings within specific adaptive windows. This highlights the method’s capability in accommodating variations in window lengths, encompassing longer, shorter, and shifted windows, independently for each signal in a pair across the entire run. This stands in contrast to conventional static windows used in SWPC, emphasizing the adaptive and dynamic nature of our approach.

Figure 3(B)highlights the poor performance of SWPC and PS in capturing the dynamics of coupled stretching and shrinking timescales, as indicated by the correlations between their estimations and respective ground truths across various window sizes for SWPC and bandwidths for PS. Conversely, the notably high correlation of 0.91 between the ground truth of coupled stretching and shrinking timescale dynamics and the estimations provided by warp elasticity underscore its reliability in accurately estimating these dynamics. Another noteworthy observation is the presence of transient anticorrelations and negative phase synchronization in the SWPC and PS estimations depicted inFigure 3(B). Various factors, such as hemodynamic delay, global signal regression, and physiological noise, have been reported to explain the occurrence of negative correlations between brain region pairs. In this study, we demonstrate that one effect of the stretching and shrinking dynamics is the emergence of transient anticorrelations in SWPC and transient negative synchrony in PS. Traditional measures often fail to account for these transient phenomena, posing a risk of false estimations of trFC and trFNC. However, warp elasticity proves capable of capturing the stretching and shrinking of coupling timescales, thereby avoiding false anticorrelations and negative synchronizations that may arise due to the stretching and shrinking effects of coupled timescales.

The stretching of brain network coupling relative to others may imply a prolonged or delayed neural and hemodynamic response within a specific brain network, indicating that it takes longer for this network to reach its peak activity or return to baseline compared with another network. Conversely, shrinking may suggest a faster response or a more rapid decay in neural and hemodynamic activity within one network compared with another. It is crucial to highlight that the HRF of brain regions can vary significantly, not only among different brain regions but also among individual subjects (Aguirre et al., 1998). Failing to consider this HRF variability can lead to inaccurate FC estimations (Rangaprakash et al., 2018). Our approach effectively addresses this inherent variability and additionally captures dynamic changes that might be related to neural or hemodynamic factors, via stretching and shrinking of timescales within dynamic windows. Another interpretation is that the warp elasticity may enable different brain regions or networks to process information at varying temporal scales relative to one another. This variation could stem from neural effects, or it could also be associated with dynamically changing hemodynamic effects, which are complex and typically not thoroughly studied.

To ensure the reliability of our measure in the presence of noise, we rigorously evaluated the sensitivity of the warp elasticity method to varying noise levels within the reported range of tSNRs in fMRI data. The results of our assessment demonstrate the robust performance of our method in the presence of noise. Across 100 samples of noisy fMRI signals at different tSNR values, we computed the average correlation of warp elasticity between these noisy fMRI time series and the actual noiseless fMRI time series. Remarkably, these correlations yielded coefficients that consistently fell within the range of 1 to 0.66 for the relevant fMRI tSNR values typically reported in fMRI studies, as illustrated inFigure 4(B). In addition, we investigated the relationships between simulated data with varying levels of tSNR values and their corresponding ground truths. We found that the reliable range of tSNR for accurately estimating the ground truth was approximately between 100 and 3.7 inFigure 4(C). Within this range, the correlation between the estimated values and the ground truth ranged from 0.87 to 0.71. These findings align with the outcomes reported byMeszlényi et al. (2017), who conducted a similar analysis comparing the noise sensitivity of DTW distances with correlation coefficients and observed that DTW distances were less sensitive to noise compared with correlation coefficients. These compelling outcomes highlight the warp elasticity measure as a considerably less sensitive metric to noise, underlining its reliability and efficacy for fMRI analysis.

In our evaluation of warp elasticity replicability, we utilized 200 randomly selected subjects from the HCP dataset and performed analyses across four bootstrap samples, focusing on cluster centroids derived from clustered warp elasticity. The results of our replicability assessment are presented inFigure 5. InFigure 5(B), we display the correlations between all four bootstrap samples across clusters. Notably, there exists a high correlation between samples 1, 2, and 3, with a minimum correlation coefficient of 0.85 observed between samples 3 and 2 in cluster 2. This high correlation signifies that samples 1, 2, and 3 exhibit a robust and consistent warp elasticity, indicating a high level of replicability among these random bootstrap samples. On the other hand, sample 4 demonstrates a comparatively lower level of correlation when compared with the rest of the samples. It exhibits a minimum coefficient of 0.65 between samples 4 and 2 in cluster 2, indicating a slightly less correlated warp elasticity for sample 4. However, given the nature of bootstrap analysis, such variability is expected. Nevertheless, these results remain convincing, underscoring the highly replicable nature of warp elasticity across randomly selected subjects.

Our analysis extended to examining how warp elasticity could capture group differences between individuals with SZ and HC. We clustered the results of warp elasticity for both SZ and HC subjects into three clusters, as illustrated inFigure 6. One intriguing discovery from our group analysis was the striking resemblance between the WE states observed in the group difference analysis and those identified in the bootstrap analysis. Specifically, in the group analysis illustrated inFigure 6, there is a notable similarity between cluster 1 of the group analysis (Fig. 6) and the bootstrap analysis (Fig. 5). Likewise, cluster 2 of the group analysis (Fig. 6) exhibits striking similarities with the bootstrap analysis (Fig. 5). This alignment between WE states across different analyses further underscores the robustness and consistency of the observed patterns of warp elasticity.

Another striking observation revolves around the consistent range of stretching and shrinking observed in both the bootstrap and group analyses. Notably, the warp elasticity estimates in the group analysis fall within the range of −0.2 to +0.2 seconds, while in the bootstrap analysis, the range extends from −0.28 to +0.28 seconds. Despite the consistency in the range of the warp elasticity between the HCP and fBIRN datasets, there exists a slight difference of ±0.08 seconds. This disparity is speculated to be influenced by several factors, including variations in the age groups of the datasets, the number of subjects included, and potential differences in the time frame of the scans. The group analysis was conducted using the fBIRN dataset, comprising 311 subjects (including both healthy control and SZ individuals), 159 time points, and a mean age of 38.77 ± 11.63 years. Conversely, the bootstrap analysis was based on the HCP dataset, which included 827 subjects, 1200 time points, and a mean age of 28 ± 6.5 years. This subtle difference in the range of warp elasticity between the two analyses may be attributed to the age discrepancy. Specifically, the younger brains in the HCP dataset may demonstrate more pronounced stretching /shrinking compared with the relatively older brains in the fBIRN dataset. Additionally, the variation in time points might contribute, as individuals in the HCP dataset might exhibit more extensive stretching/shrinking due to an extended scanning period compared with those in the fBIRN dataset. Moreover, the difference in the number of subjects could potentially contribute to more generalized estimations in the HCP dataset, as it provides a broader range of subjects compared with the fBIRN dataset, which has a smaller subject pool. It is important to highlight that despite the slight difference in the range of warp elasticity values, the overall patterns between the group and bootstrap analyses remain consistent.

InFigure 6, the WE states appear similar between patients with schizophrenia and controls. However, significant differences emerge in the dynamics of these WE states, particularly in terms of their mean dwell times, transitions, and occupancy. This could be attributed to the possibility that stretching and shrinking patterns constitute a fundamental neural dynamic, unchanged between these two groups. Coupled with the similarity of the group analysis WE states to that of the bootstrap analysis, these WE states strike an interest in further speculations of what they could potentially mean. Cluster 1 inFigures 4and5shows stretching of CC and DM networks with respect to all other networks SM, Aud, Vis, Cb, and SC regions. The stretching observed in the CC and DM networks during resting-state scans suggests that there might be an increased level of cognitive engagement or activity at certain times. These networks play a role in higher-order cognitive processes such as self-reflection, mental planning, and executive control. The stretching could reflect fluctuations in the degree to which individuals engage in these cognitive activities while at rest. Furthermore, stretching in the Vis networks relative to SM, Aud, Cb, and SC, particularly in cluster 1 of sample 4 of the bootstrap analysis (Fig. 5), is also evident although it is not as pronounced as in the CC and DM regions. This stretching may indicate that the Vis networks are working in coordination with the DM and CC networks during periods of mind wandering and mental imagery. The literature suggests evidence of mental imagery being involved with Vis networks (Ganis et al., 2004;Le Bihan et al., 1993;Pearson, 2019). It is noteworthy to mention that, as shown inFigure 7, the fraction rate of this state is the highest compared with other states, indicating that it is the most prevalent state. This aligns with our expectations regarding the heightened activity of DM and CC regions during rest.

Cluster 2 exhibits stretching in the Vis networks with respect to all other networks in both the group analysis and bootstrap analysis. This observation might suggest a WE state characterized by heightened mental imagery or visual thought processing during resting-state scans. While the resting state is typically associated with the default mode network, the emergence of this state with unconventional results is not entirely unexpected. This is because we know that imagination and mind wandering are common during resting state. We speculate that the Vis networks could potentially play a more significant role in mind wandering and imagination than previously thought, given their stretching relative to all other brain networks (Ganis et al., 2004;Pearson, 2019). It is important to note that this interpretation is speculative and based on prior knowledge.

Cluster 3 presents an intriguing inverse pattern compared with Cluster 2, where the Vis networks appear to shrink relative to all other networks in the group analysis. Notably, this pattern is also primarily observed in sample 4 of the bootstrap analysis within cluster 3. This raises a significant question, as per our previous speculations. It would imply a state where Vis networks are exhibiting lower processing activity in comparison with all other networks. The implications of this state are uncertain and warrant further investigation. Another noteworthy aspect of this state that aligns with the bootstrap results is the stretching of Cb networks with respect to all other networks. This finding hints at the potential involvement of cerebellar regions in higher cognitive processes, which is consistent with prior studies (Adamaszek et al., 2017;Beuriat et al., 2022;E et al., 2014;Koziol et al., 2014;Van Overwalle et al., 2020). These studies have indicated that cerebellar regions play a role in coordinating cognitive tasks such as timing and sequencing.

We detected a statistical difference between SZ and HC groups in cluster 2’s mean dwell times, following FDR-corrected p-values obtained from a two-sample*t*-test between SZ and HC for the clusters highlighted inFigure 7(A). Notably, HC subjects spend significantly more time within cluster 2 (a state that suggests mental imagery and visual thought processing) once they enter it compared with SZ subjects. Similar significant differences were found for cluster 2 in the fraction rate, as depicted inFigure 7(B). HC subjects also spent significantly more time in cluster 2 than the SZ control group. Furthermore, we identified significant differences between individuals with SZ and HC in cluster 1 based on the fraction rates of the two groups. In this case, the group fraction rate for SZ was found to be significantly higher than that of the HC group, meaning SZ groups spend longer time in a state that suggests higher-order cognitive processing such as DM and CC processing over HC at rest. Lastly, the examination of transition matrices reveals significant differences in transitions, specifically (2→1), and (2→2) transitions, as illustrated inFigure 7(C). These findings align with the results obtained from mean dwell times and fraction rates and provide further insights. The (2→2) transition indicates a significantly higher probability of HC remaining in WE state 2, which is associated with visual thought processing, compared with individuals with SZ. Specifically, HCs are more likely to stay in this visual processing state than SZ. Conversely, the (2→1) transition highlights a significant difference in the likelihood of subjects with SZ transitioning from WE state 2, linked to visual thought processing, to state 1, which is characterized by mind wandering and higher levels of cognitive processing. This transition is more probable for SZ subjects than for HCs, as demonstrated inFigure 7(C). These findings suggest distinct differences in cognitive processing patterns and state transitions between HC and SZ groups.

The observed disparities in stretching and shrinking dynamics of brain region coupling between individuals with SZ and HC are intriguing, yet the exact implications remain uncertain. It is evident, however, that these differences exist, potentially stemming from the intricate interplay of neural and hemodynamic factors. This complexity underscores the need for further exploration, particularly through the application of advanced dynamic programming algorithms such as DTW.

In our study, we utilized warp elasticity to estimate the stretching and shrinking of signal coupling within specific adaptive windows, as illustrated inFigure 1. While this approach provided valuable insights, it is important to note the limitations. The visualization of stretching and shrinking windows in the actual fMRI time series pair was not feasible due to the intricate and multifaceted nature of these phenomena. Moreover, the precise significance of these stretching and shrinking patterns within interconnected brain networks remains elusive. To offer some intuitive understanding, consider cluster 2 inFigure 6as an example. It suggests that the Vis networks may stretch approximately 6 seconds in a 30-second timeframe (which is 0.2 times 30 seconds) in relation to CC and DM networks. Despite these visualization and interpretative challenges, our research results are both intriguing and hold great potential. The intriguing results we have obtained serve as a catalyst for further investigations aimed at unraveling the nuanced meanings underlying connectivity alterations. Subsequent research may benefit from incorporating further clinical metrics or analysis of specific subgroups to delve deeper into how chronic mental health conditions, disabilities, and the usage of psychotropic medications may affect the observed patterns of the dynamics of the timescale of functional connectivity within the schizophrenia cohort. Also, we suggest that further studies perform warp elasticity on EEG data as this may reveal more granular stretching and shrinking patterns considering the high temporal resolution it provides. Warp elasticity could enhance our grasp of the underlying influences on the findings and their relevance to clinical endpoints. This study not only highlights the existence of these dynamics but also emphasizes the imperative to delve deeper into their implications, driving us toward a more comprehensive understanding of the intricate workings of the human brain.

## 5 Conclusion

In conclusion, our study delves into the complexities of brain network dynamics using the innovative warp elasticity method, revealing intricate patterns of stretching and shrinking of brain region coupling or connectivity within specific adaptive windows. By employing advanced techniques such as DTW, we highlighted the adaptability of brain regions over time, shedding light on the temporal nuances often overlooked in conventional analyses. Our research, conducted with rigorous methodology and thorough evaluations, underscores the robustness and reliability of warp elasticity in capturing these dynamic changes, even amidst the challenges posed by noise and individual subject variability. The observed differences in stretching and shrinking dynamics in the coupling of functional connectivity between individuals with schizophrenia and healthy controls, although intriguing, pose compelling questions about their underlying neural mechanisms. While our study marks a significant step forward, further research is imperative to unravel the precise implications of these findings, offering a promising avenue for advancing our understanding of the intricacies of human brain connectivity.

## Data and Code Availability

The function for estimating the warp elasticity in MATLAB language can be accessed through GitHub (https://github.com/Sirlord-Sen/DTW_warp_elasticity). The data were not collected by us and were provided in a deidentified manner. The IRB will not allow sharing of data or individual derivatives as a data reuse agreement was not signed by the subjects during the original acquisition.

## Author Contributions

Sir-Lord Wiafe: Conceptualization, Formal analysis, Methodology, Visualization, Writing–original draft. Ashkan Faghiri: Conceptualization, Methodology, Supervision, Validation, Writing–review & editing. Zening Fu: Resources, Writing–review & editing. Robyn Miller and Adrian Preda: Writing–review & editing. Vince D. Calhoun: Conceptualization, Funding acquisition, Validation, Methodology, Resources, Supervision, Writing–review & editing.

## Declaration of Competing Interest

None.

## Supplementary Material

Supplementary Material
