# Supplementary material for: The dynamics of dynamic time warping in fMRI data: A method to capture inter-network stretching and shrinking via warp elasticity
Source: Imaging Neurosci (Camb). 2024 Jun 3;2:imag-2-00187. doi: 10.1162/imag_a_00187 (PMC12247588; doi:10.1162/imag_a_00187)
Supplement: Supplementary Material [file imag_a_00187-supp.pdf]

Bootstrap analysis

In the bootstrap analysis aimed at demonstrating the robustness of the warp elasticity, we employed a method of random selection. Specifically, we randomly selected four sets of 200 subjects from the HCP dataset and computed the warp elasticity for each bootstrap set independently. Below is the elbow plot illustrating the warp elasticity for one of these bootstrap sets.

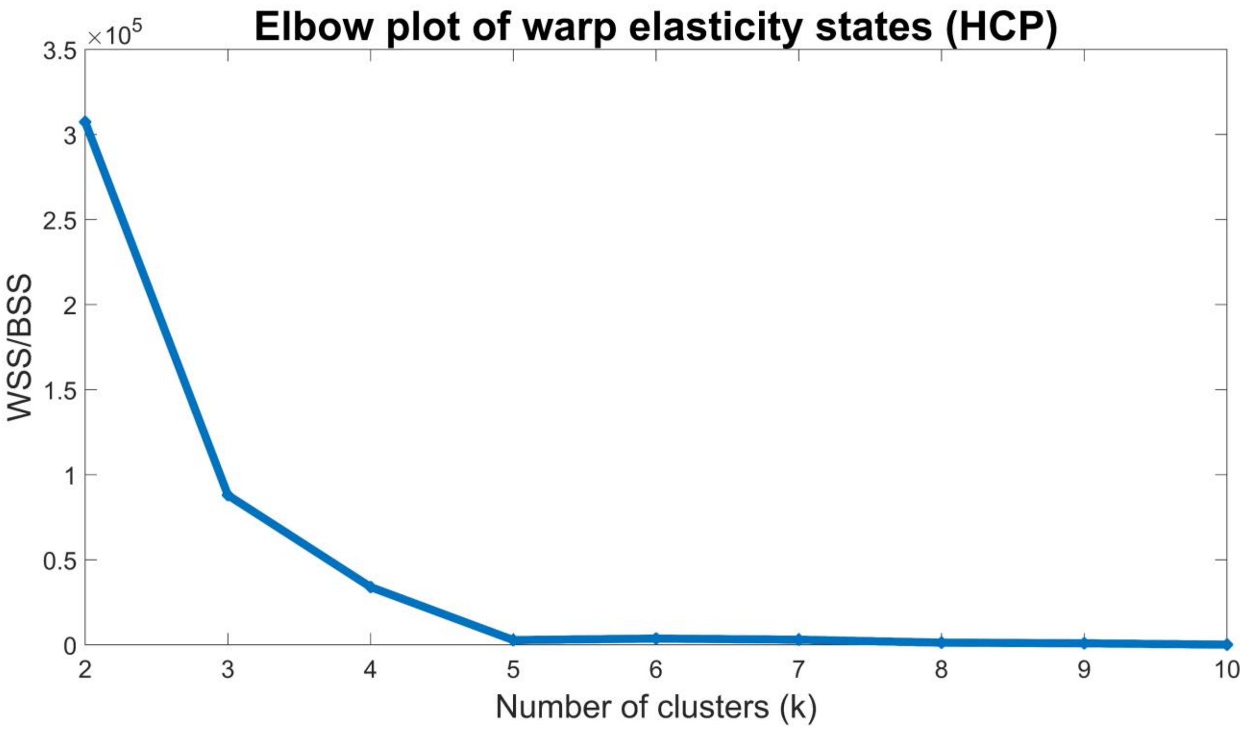

Figure 1. Elbow plot of warp elasticity clusters from the human connectome project (HCP) dataset for bootstrap analysis.

Group analysis.

Kmeans clustering is performed on the warp elasticity of the fBIRN dataset to obtain recurring stretching/shrinking patterns in the whole brain activity. The elbow plot is shown before.

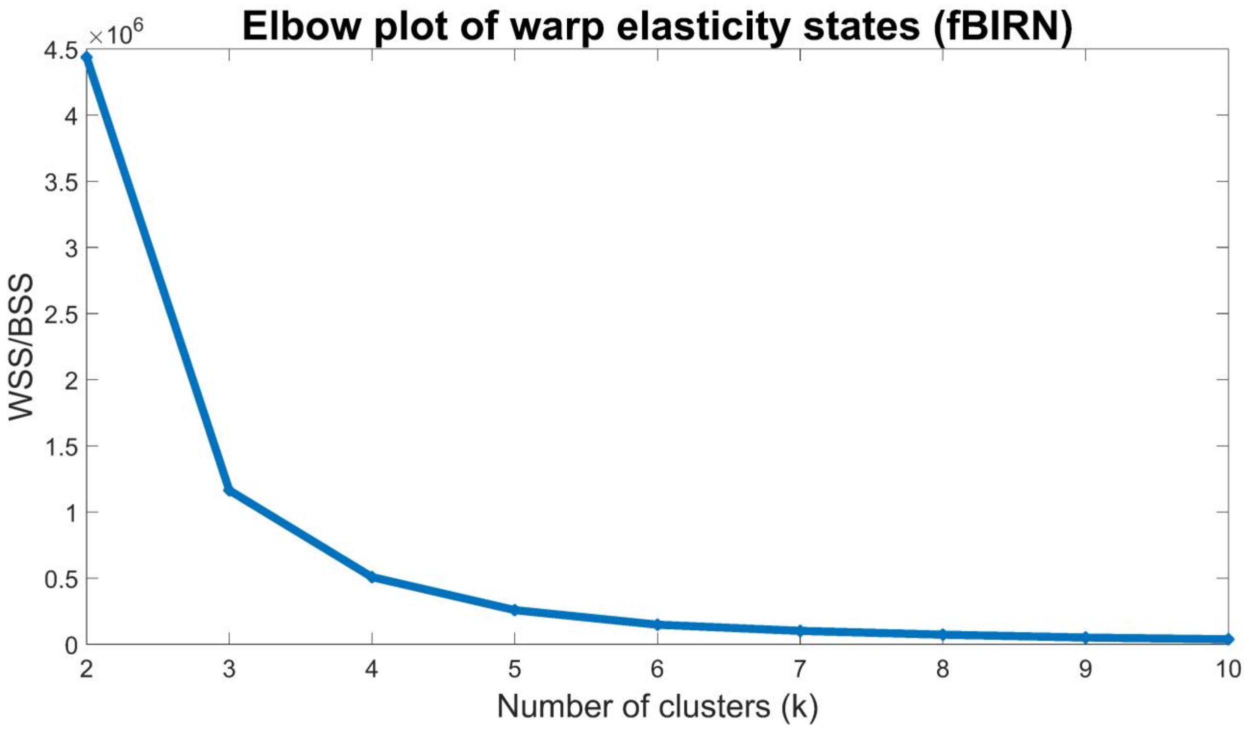

Figure 2. Elbow plot of warp elasticity clusters from the Function Biomedical Informatics Research Network (fBIRN) dataset for group analysis
